# A Process Evaluation to Assess Contextual Factors Associated With the Uptake of a Rapid Response Service to Support Health Systems’ Decision-Making in Uganda

**DOI:** 10.15171/ijhpm.2017.04

**Published:** 2017-02-04

**Authors:** Rhona Mijumbi-Deve, Nelson K. Sewankambo

**Affiliations:** ^1^ Clinical Epidemiology Unit, School of Medicine, College of Health Sciences, Makerere University, Kampala, Uganda.; ^2^ Regional East Africa Community Health Policy Initiative (REACH-PI) (Uganda Country Node), Makerere University, Kampala, Uganda.; ^3^ Department of Medicine, School of Medicine, College of Health Sciences, Makerere University, Kampala, Uganda.

**Keywords:** Evidence-Informed Health Policy, Knowledge Translation (KT), Rapid Response Services (RRSs), Innovations, Process Evaluations

## Abstract

**Background:** Although proven feasible, rapid response services (RRSs) to support urgent decision and policymaking are still a fairly new and innovative strategy in several health systems, more especially in low-income countries. There are several information gaps about these RRSs that exist including the factors that make them work in different contexts and in addition what affects their uptake by potential end users.

**Methods:** We used a case study employing process evaluation methods to determine what contextual factors affect the utilization of a RRS in Uganda. We held in-depth interviews with researchers, knowledge translation (KT) specialists and policy-makers from several research and policy-making institutions in Uganda’s health sector. We analyzed the data using thematic analysis to develop categories and themes about activities and structures under given program components that affected uptake of the service.

**Results:** We identified several factors under three themes that have both overlapping relations and also reinforcing loops amplifying each other: Internal factors (those factors that were identified as over which the RRS had full [or almost full] control); external factors (factors over which the service had only partial influence, a second party holds part of this influence); and environmental factors (factors over which the service had no or only remote control if at all). Internal factors were the design of the service and resources available for it, while the external factors were the service’s visibility, integrity and relationships. Environmental factors were political will and health system policy and decision-making infrastructure.

**Conclusion:** For health systems practitioners considering RRSs, knowing what factors will affect uptake and therefore modifying them within their contexts is important to ensure efficient use and successful utilization of the mechanisms.

## Introduction

Over the past few years, researchers in low- and middle-income countries have shown the feasibility of rapid response services (RRSs) to meet policy-makers’ urgent needs for research evidence about health systems to aid policy-making in low income countries.^1,2^ RRSs are designed to receive policy and decision questions and respond to these with the best available research evidence in summarized and contextualized forms within short periods of time, for example, less than 28 days.^1^

A framework to assess country-level efforts to link research to action cites RRSs as a form of ‘facilitating user-pull’ effort – a strategy to facilitate efforts initiated by policy-makers to access the best available evidence – through approaches like written summaries.^3^ These RRSs improve the timeliness and relevance of research evidence for policy-making. They are also thought to improve contact and interaction between users and producers of research evidence, the lack of which has been cited as a barrier for the use of research evidence during policy and decision-making.^4,5^ The Ugandan country node of the Regional East African Community Health - Policy Initiative (REACH-PI), a knowledge translation platform and a partner in the Supporting Use of Research Evidence (SURE) for Health Policy in African Health service and systems project, piloted a RRS starting in March 2010 and showed that RRSs were not only feasible but also welcome to policy-makers, who indeed valued the services and the products.^1,6,7^

Implementers and researchers alike have limited experience with these RRSs. RRSs are still a fairly new and innovative strategy in several health systems, more especially in low-income countries, and like with many new innovations, there are still several information gaps about them. At the time of this research we did not know of any service that had been systematically evaluated in a low-income country to learn how and why it works.

The knowledge on why and how the RRS works are important to inform among other things, what affects its uptake by potential end users. (The concept of ‘Uptake’ as used in this paper refers to the action of making use of something that is available). Although their invention or development may take a shorter and more straightforward course, the uptake of innovations is usually slower in comparison.^8^ The eventual uptake of a program is a result of several smaller decisions comparing the benefits and costs of adoption of the innovation, all of these happening in an environment of uncertainty.^9^ Individuals and institutions will tend to take up innovations when the value or benefit they put on them outweighs the costs. And in many cases this is influenced by many factors, some of which may be standard while others context-sensitive.

Context in knowledge translation (KT) is a subject that has attracted a number of theories and a growing body of literature, underscoring its importance.^10-16^ For example, Kitson and colleagues argue that successful implementation of research into practice is a function of the interplay of three core elements including the context into which the research is positioned.^10^

Context is important in, and for KT. Contextual factors are perceived to be significant barriers to research utilization and related activities,^17^ including strategies and platforms to support these. Although, contextual factors affecting the uptake of KT strategies have not yet been well articulated – indeed we do not know of any study that has looked at factors affecting the uptake of a RRS in any setting – several factors affecting the uptake of evidence for policy and practice have been documented. Scholars have cited factors in the context of organizations and systems that affect uptake of innovations as including the strength of the relationship between the producers or suppliers and the end users, including the importance of network effects.^9^ Other factors include culture referring to the norms and values of the environment in which the KT is placed, and power (perceived and/or actual).^18,19^ Innovations will tend to be taken up and achieve higher levels of saturation if they have among other things, high compatibility with current norms and work processes.^20^ Other influential factors include internal organizational structure – centralization, complexity, formalization, interconnectedness, organizational slack, and external characteristics of the organization.^15^ Furthermore, the customs and behavior of the supply side, affect and sometimes facilitate or act as a barrier to the eventual acceptance and uptake of the innovation.^8^

It is important to note that both the user and supplier of the innovation may have no direct control over some contextual factors within the environment or institution that the innovation is being introduced. Such factors may include institution rules and culture, government regulations, and country economic situations.^15,18,19^

Despite the work done thus far, scholars still need to understand more on the concepts of context in KT. Kimberly and Cook emphasize the importance of understanding context domains from not only theoretical but also practical standpoints.^16^ They categorically point out that lack of conceptual clarity about context is one of the major contributors to difficulties in interpreting studies of KT activities. This study aimed at exploring the contextual factors associated with the how and why an RRS may be taken up by users in Uganda. Such factors are important for, and should be considered during the implementation and scale up of RRSs in similar settings.

## Methods

### Design

We used a case study employing process evaluation methods.^21-23^

### The Case

The case or unit of analysis in this study was the RRS at the College of Health Sciences in Makerere University. The study period is between March 2010 and May 2014. The RRS is designed to receive, and respond to urgent requests for research evidence about health systems from policy and decision-makers. The service although acknowledging the importance of all other elements of health care especially clinical practice, defined its scope to include themes of health systems, for example, governance, delivery arrangements and financial arrangements.

Structure: The service was based at Makerere University, the oldest and largest university in Uganda located in Kampala, the capital of the country. The program components of the service included goals and objectives, client/target group, personnel, equipment, finances, operations, and products. The service was coordinated by staff who were supported by a wide network of researchers in and outside the region. The staff would receive questions from the policy-makers by telephone, email or physical contact. They would then take the policy-maker through a process of clarifying the question to ensure that the question was not only clear and asked in an answerable manner, but that it indeed fell within the scope handled by the service. (Requests were rejected when they did not fall into the scope of the service in terms of topic or theme or urgency [where information was needed in more than 28 days]. In such cases the policy-makers were politely directed to a source that the researchers felt would best handle the question). Following this they searched for research evidence relevant to this query, appraised it, contextualized and summarized it. This summary would then be reviewed by local and international experts on the given subject. Such experts were identified through several processes – they could be authors from the literature reviewed, or experts identified by senior researchers on the study or through colleagues in other institutions like the World Health Organization (WHO). Once the review process was complete, the staff prepared a short brief of usually four pages maximum, with clear key messages which would be submitted to the policy-maker. (Where the available time was short, for example, with responses required in less than 5 days, an internal review was done by a senior researcher on the service. The brief would still be sent to an external reviewer and the policy-maker informed that if the reviewer’s input was substantially different from the brief delivered to him/her, an updated version would be provided as soon as possible). The above process would take any time less than 28 days. The service began with its scope limited to health systems questions concerning organizational arrangements, governance arrangements, strategies for implementing change, and financial arrangements. After the first 6 months, the scope was widened to include questions about health technology assessments.

At the designing stage of the service, we imagined that such a service would benefit mid to top level policy and decision-makers at ministries of health, districts or local governments, Civil Society Organizations, health-related multi-, and bi-lateral agencies, the private sector, and legislators like parliamentarians among others. Such decision-makers would not only have to be involved in making urgent policy decisions regularly but they would also have to recognize and value research as an input in the policy and decision-making process. We took the following steps in developing the structure: (*i*) reviewing the literature around rapid response mechanisms similar to that proposed; (*ii*) brainstorming what the literature revealed; and (*iii*) then using this information to design a RRS. This design was presented to potential users (policy-makers) through consultative interviews and their feedback used to modify it before piloting it.

### The Context

The RRS pilot begun in March 2010, a time that is seen as a turning point in the economic and political and social contexts of the country.

*Sociopolitical context*: 2010 was a time when there was significant desire and enthusiasm for developing new, and reviewing health policies in place. Debates in the Ministry of Health (MoH) and parliament were intense and frequently pushed politicians and policy-makers to require information urgently. The leadership in the MoH was also significantly intent on using evidence for decisions as was seen in several of the ministry’s Technical Working Groups and individual leaders pushing for the practice. Indeed the director of planning at the time requested for a brief on how to improve the use of evidence in decision-making and policy development in the ministry. Several decisions that had already been taken or were in the process were repealed when the evidence was against them. On the wider national level, Uganda was preparing for an election in the next year. Election periods usually raise the profile of health and health decisions in the country. Vital decisions are considered then. For example, debates around user fees, national health insurance and others have escalated during these times.

*Health policy context:* At the time of this study, the major policy formulating body was the MoH. The sector partners were and are active and influential actors in the policy-making process. These include development partners and civil society too. Actors like managers at the district should also be as active but are often left to implement central policies and decisions despite the system being decentralized. The different actors represent different interests and agenda which may or may not be in line with those of the government or the MoH.This ends up affecting the content of policies and decisions, depending on the overriding or more influential interest. Notably too was the high turnover of top management and administration in the MoH as the major policy-making body, which directly affects the process, context and content of policies and decisions. Furthermore, at the time of this study, many of the sectoral decisions and institutions were vertically oriented; that is, programs aligned with diseases run parallel to each other and there was less attention paid to horizontal systemic issues. This in many ways continued to weaken the system as a whole yet strengthen parts of it causing an imbalance that affected several aspects like human resources.

*Economic:* Economically, 2010 was the height of a decade of high economic growth rates. From 2003 till then, Uganda’s annual gross domestic product growth rate was at 7.3%.^24^ This plunged majorly to 3.5% in the next year and has remained below 5% since. The high growth rate at the time was attributed to a dynamic service sector. Significantly too in 2010, the first national development plan (NDP) 2010/2011–2014/2015 was launched with the intention of moving away from short-term and sectoral-specific planning and towards a synchronized and holistic approach to development planning intended to deliver long term development aspirations of the country.^25^ There was and continues to be an effort for policies, decisions and programs to align themselves with the NDPs, seeking data, information and evidence frequently on what and how to do this. This noble planning has however been plagued with poor accountability and corruption, having in fact worsened after 2008.

### Data Collection

We involved researchers and KT specialists who had either worked on or with the RRS during the pilot and scale up period. In addition, we involved participants from several research and policy-making institutions in Uganda’s health sector including a variety of informants from mid-top level policy-makers at the MoH, districts, Civil Society Organizations, bi- and multi-lateral agencies with varied backgrounds, who identified themselves either as researchers (including KT specialists) or policy-makers. These participants had been involved in using the RRS and/or were conversant with its operations. We purposively sampled from these until we had reached data saturation.

We collected data using in-depth interviews that lasted approximately 1 hour, using an interview guide whose questions were open-ended (the questionnaire and interview guide are attached as Supplementary files 1 and 2, respectively). The interviews were both face-to-face and by skype calls. These interviews were conducted by the principal investigator of the study, over a period of 8 months. The interviews were transcribed immediately.

We identified the components that make up the RRS and contribute to the completion of its process.^22,23^ The participants were asked to reflect on the RRS in this study and how it faired on these components, and how they might have affected uptake of the service. Participants were prompted to talk about structures and activities under the components, so as to get to the ‘why’ and ‘how’ of each respectively.

### Data Analysis

We analyzed the data using thematic analysis, going through three stages: line-by-line coding of transcripts; organizing the ‘free codes’ into related areas to construct descriptive categories; and developing categorical themes. We coded each line of text according to its meaning and content using inductive coding. Codes were structured as ‘free’ codes, that is, without a hierarchical structure. These codes were then organized to explore and show relationships if any, to develop descriptive categories not only of why the category was important but how it was. Any patterns and connections within and between these categories were further explored. Different thematic categories were developed from which we drew conclusions, establishing and describing the most important determinant factors for the uptake of RRSs in Uganda.

Prior to collecting data, we considered the need to balance our roles as the initial implementers of the RRS and then evaluators during this research – that is, the role of the participant observer. Using a subjectivist’s lens which argues that the involvement of the researcher should be actively encouraged, our focus on the meaning of the social phenomena and our goal was to understand and explain what we saw in its contextual setting.^26^ We used our opinions to inform the planning process and the initial data collection process. For example, we used our knowledge and opinions to identify the program components to be explored which is a major first step of a process evaluation. In addition, we used the participants’ responses to corroborate and formulate a fuller picture.

We obtained ethical clearance for this study from the School of Medicine Research and Ethics Committee at Makerere University. We also sought written free and informed consent from each of the participants before the interviews.

## Results

We interviewed 21 respondents whose profile is provided in Table. We interviewed an almost equal number of researchers who include KT specialists and policy-makers and most of these respondents were either affiliated to a university (7/21) or the Uganda government MoH (6/21).

**Table:** Profile (Self-reported Attributes) of Participants Interviewed

**Attribute**	**No. (%)**
Identification	
Health systems and KT researcher	11 (52)
Policy-maker	10 (48)
Institution of affiliation	
University	7 (33)
Research institution	2 (9.5)
WHO (Country office)	2 (9.5)
MoH	6 (29)
Development partner agency	2 (9.5)
Civil society	2 (9.5)
Gender	
Male	14 (67)
Female	7 (33)
Age group	
30-39	3 (14.3)
40-49	13 (61.9)
50-59	3 (14.3)
60-69	2 (9.5)
Years as researcher/policy-maker	
<5	1 (4.8)
5-10	4 (19.0)
11-15	9 (42.9)
16-20	5 (23.8)
>20	2 (9.5)

Abbreviations: WHO, World Health Organization; MoH‏, Ministry of Health; KT, Knowledge translation.

From the participants’ responses emerged several thematic and descriptive categories as represented in Figure.

**Figure:**
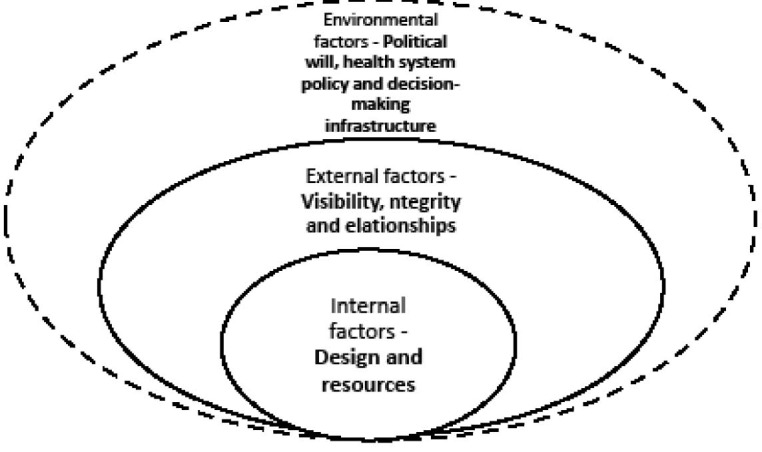
Thematic and Descriptive Categories in an Overlapping Relationship as They Emerged From the Process Evaluation of the RRS Piloted by REACH-PI (U) in Uganda. Abbreviations: RRS, rapid response service; REACH-PI, Regional East African Community Health - Policy Initiative.

### Thematic Categories

There emerged three thematic categories: internal factors, external factors and environmental factors. Internal factors refers to those factors that were identified as over which the RRS and its management had full (or almost full) control. External factors were identified as factors over which the service’s management had only partial influence while environmental factors were those factors over which the service had no or only remote control if at all.

There are several simple and complex relationships between these categories and factors. Although they are presented as mutually exclusive thematic categories or factors, they are in fact inter-related in many ways and are not independent of each other. The simplest relationship is that of an overlapping one as represented in Figure. That is, the internal factors shape the design of the mechanism directly; the external factors then act as a second layer of shaping of the internal factors and therefore of the mechanism. The service is complete with the first layer but only structurally while the second layer makes its activities (in addition to the structure) possible and complete. The two layers make a functional mechanism. The third layer creates the environment in which it operates, further molding it.

In a more complex relationship, although the categorical themes are shown to have a direct influence on uptake, there are also interactive relationships between the different descriptive categories which would eventually impact uptake albeit through longer indirect routes. For example, relationships between the supply and the demand is thought to have a direct influence on the uptake of the RRS. In addition, an increase in resources like supply side human resources would improve chances of uptake. Yet relationships may also improve access to resources like financial resources or reviewers, which improves the design of the service, eventually improving chances for its uptake. The same can be shown for several factors within and across the three thematic categories.

### Descriptive Categories

#### Internal factors – Factors That the Service Has Full or a Majority Control Over

##### Design

The design of the service emerged consistently as an important factor affecting its uptake. Part of the design is the goals and objectives of the service which respondents noted should tally with the *needs of the users*. One respondent, a policy-maker, pointed out how the service should have a clear demonstration of an objective to fill a gap that is clearly perceived as such by the users.

It is important too to incorporate in the design mechanisms to remain aware of the needs of users, which in this case was through a constant consultative process before and during the service’s implementation. For example, one researcher noted that it was important that the service was not thought out and designed by researchers alone but that there was *consultation with policy-makers* in the form of interviews at the start and an advisory board throughout its operations. This ensured relevance to the potential users, continued consideration for their needs, a feeling of ownership and enhanced understanding within the users, factors which in turn increased chances of uptake.

*“Uhmm so I guess what might help to sort of address the disadvantage of not being in the ministry is … an advisory board or eventually a steering committee … people in your target audience, people in the ministry in the advisory group …”*(Researcher).

Designing a service that worked within the current *norms and behavior of users* was an important factor. Respondents noted that the service did not use measures that were too drastically different from what the users were accustomed to but incorporated proactive behavior change mechanisms like advocacy, sensitization and reminders. The RRS used regular reminders and email alerts, plus short presentations in policy-makers’ meetings, to persuade them use the service to support their usual decision-making process. This, the respondents felt would have been more persuasive leaving a feeling of ‘safety.’

One researcher noted that the simplicity of the design of the service from the users’ point of view (both actual and perceived) was important for uptake. This was in reference to the fact that the RRS being studied here was designed to ensure that users could rapidly contact the service, going through only one step. For example, it only took a phone call, email message or physical contact, to have immediate interaction with the service.

*“Quick and easy access, so people can contact within seconds, doesn’t take any effort, either by email or a phone, there is no hassle. Quick follow up to clarify the question and whether it’s a relevant question for the service or not …”* (Researcher).

In clarifying the advantage of simplicity, the researcher gave an example of a call center that has the caller transferred through a number of steps or wait in line before they can access someone to talk to.

Another factor affecting uptake was the *balance between the demand and supply* sides with a view to moderate users’ expectations and meet these appropriately. Several respondents identifying themselves as researchers and were conversant with the REACH-PI RRS’s design and implementation processes noted how this was done. They noted that the service started with only a few users who understood the program and what it was about, expanding gradually as they studied and adjusted to the demand and the capacity available. This ‘tapered’ start ensured that the supply side was not overwhelmed by the demand side of the service, while the demand side was not disappointed with promises that could not be met.

*“… pilot testing things and reflecting on what was working well as you scaled it up, you know from the outside it just seemed like an incredibly thoughtfully planned out scaling up process. So instead of announcing tomorrow that we are ready to go, we are going to do twenty rapid responses a year, clearly you did some, reflected on them and then moved on to another set, and then reflected on them …”* (Researcher).

Providing opportunities for feedback within the design of the service through follow-up interviews after every report was delivered and availing contacts on every report written by the service, was a factor thought to improve uptake of the RRS. The opportunities not only allowed for users to express their needs and concerns immediately, it also allowed for the researchers to respond in a timely manner and correct any issues going wrong, hence making themselves relevant and desirable to users.

One policy-maker noted that the service endeavored to be ‘personal’ which made it desirable. This was echoed by a researcher working on the service who noted that much as policy-makers can be taken as a group, they are still individuals with unique characteristics. A service that endeavors to know its users and meet some personal needs within the general climate of the service delivery is likely to be taken up much more than a one-size-fits-all service.

One respondent noted that the design of the monitoring and evaluation component of the service was also helpful in increasing its uptake. She noted that the fact that this was designed around user satisfaction while many KT initiatives’ success is measured on whether the products were used in any way and pay little attention or none at all to user satisfaction, was a major advantage. Where users are satisfied uptake will increase and so it is important to monitor that.

*“… intended for the policy-maker, it is important to check in with them regularly to see how satisfied they are … not where you are counting your own score of documents used …”* (Policy-maker).

*“You also had, I believe, explicit user-testing of things … my sense was that this is a kind of wonderful case study of what it means … to plan the scale up in a very sort of staged way where you learn at each step through user testing and feedback from clients and then adjust as necessary …”* (Researcher).

##### Resources

Respondents pointed out in different ways how and why resources would affect uptake. Using words like ‘*assets,*’ ‘*inputs*’ and ‘*means to sustainability,*’ respondents noted that the amount of, and the nature of resources on the service would affect its capacity and therefore how much it can reach the potential users. One policy-maker noted that all the resources may be looked at as assets but would in fact become liabilities if appropriate attention was not paid to them. He gave an example of a service whose uptake might already be on the rise but gets ruined because of having an unqualified human resource.

#### Three major resources were identified – human resources, financing, and time.

##### Human Resources

All respondents agreed that one major resource that greatly determined the uptake of a RRS was the human resource more especially on the supply side. How qualified and skilled these are may determine whether the service produces quality and credible work which in turn gives users confidence to utilize the service. Such qualifications and skills included knowledge not only of research but more so of the policy-making process. Human resources with good social skills were thought to have a good influence on uptake of services. Furthermore human resources with good communication skills would more likely have a positive influence on the uptake of the service. The Ugandan RRS was noted for having had most of the necessary qualifications and skills.

*“… the Ugandan team was very fortunate to find someone who had all of research methods, clinical epidemiology background and health systems; there aren’t that many people …” (*Researcher).

*“Human resource is very, very important and you need to get the right people who are interested, that do not see it as simply a responsibility but have got this issue at the center of their interest” (*Researcher).

In further clarifying on how human resource contributed to uptake, quality human resource were seen as those who kept up to date with the policy issues of the day to maintain contextual relevance at any one given point in time. In addition such researchers would orientate themselves to the needs of the users.

*“… the researchers need to realize that those users have certain needs and their priority areas need to be researched into, otherwise you can go … off-target … and so it is not just researchers but researchers oriented towards the needs of the policy-makers as a critical piece”* (Policy-maker).

Other skills deemed vital were oral communication skills in addition to writing which was seen as a basic skill.

*“… so the ability to communicate very clearly and because I am assuming that sometimes people want a face-to-face presentation, also that ability to communicate effectively in a verbal way, rather than just written”* (Policy-maker).

Respondents emphasized that part of the inputs would be to raise the skill levels of human resources through training as it was unlikely that all hired persons would have the required full skill set. This training would be a secondary factor that would improve human resource which would in turn improve and maintain uptake.

*“… because I think maybe part of what is needed, I don’t think you can expect to find people who just woke up and have both of those backgrounds and so there would be training …” *(Researcher).

##### Time

Time was noted as a factor that affects uptake in two ways by allowing for the service’s maturity and in reference to the researchers having time to do what activities were required. One policy-maker noted that uptake increases with time.

*“I don’t know when you decide that it has not been taken up or that there is a delay. What I know is that the more time you give it with all things stable, the more it will be taken up. It needs time to mature, it needs time to be known; it needs time”* (Policy-maker).

The same policy-maker was quick to add that this increase would plateau out at some point in time and therefore time was also a factor in the decrease of uptake of the service. He said that at this point some of the other factors would have to be modified to keep the service relevant and for uptake to continue or even rise again.

Time was also noted in terms of ‘protected time’ for those who work on such services. Researchers on the team noted that it was important to have protected time, for example being fully employed on the service as opposed to being only available for a few hours. This, the researchers noted, was because requests are not predictable and they come in, in urgency. To be able to satisfy the end users, whose satisfaction would determine their continuing to use the service, it was necessary that researchers on the service are always available to policy-makers. This reliability would inspire confidence in them and they would take up the service much more easily than if there were times access to the service was not possible because researchers were not available to help them.

*“… having a secure base with the team so that they have a dedicated person, fulltime - also with support around you so there is a team …”* (Researcher).

##### Financing

All respondents agreed that it was impossible to have a service that would be efficient enough to inspire uptake if it lacked the financial resources. Finances were needed to ensure most of the other inputs that would make a solid and credible service.

*“… having a viable business plan … reasonably funded and sustainable … the more finances the more services and capacity you can deliver, reach more people and increase those who are utilizing you. But when you don’t have money to pay people or pay for research journal subscription, automatically you affect your quality of work and you cut down on how many you can serve. So it is kind of self-defeating where even when people want you, you can’t …”* (Policy-maker).

One policy-maker also pointed out that uptake might be higher when the financial cost of services incurred by the user is low or almost none. And so the financial barrier to the user is absent and this is attractive to users. However, he cautioned that this may also affect uptake negatively because some users tend to put a value on the service depending on what it costs and because it is free, they may value it less and even shun it.

### External Fctors

#### Visibility

Visibility was a theme that all policy-makers emphasized. The respondents noted that if the mechanism was not known to its potential users, it was impossible for it to be taken up. These policy-makers noted that although they had used or knew about the RRS, many of their colleagues did not know about it and hence might not have made use of it when they otherwise could have.

Aside from plain knowledge about the service’s existence, other activities that were noted in reference to this visibility were branding, a consistent name, clarity of goals and physical location.

In reference to branding, respondents pointed out the instant recognition provided by branding. Being able to recognize the service and its products out of many others increases chances for it to be utilized. One policy-maker cited the fact that she could recognize the rapid response branded documents from the time she first saw one with a colleague during a meeting.

A consistent name was cited for good visibility. Changing names or variability of possible names especially where a service is part of a consortium or a partnership may cause confusion to the extent that uptake is affected. Referring to the RRS in Uganda, one policy-maker pointed out the fact that on different occasions, the RRS had used names like the REACH-PI service, SURE project service, Makerere University’s service or just merely the RRS. This he noted could cause confusion and in turn affect uptake of such a service. He emphasized that a name is a brand and should be consistent.

Furthermore on visibility, respondents also cited clarity of goals and objectives of the service as part of its visibility. Referring to the RRS, one policy-maker asked what the service’s main objective was. He wondered whether the RRS was a service to the policy-makers or a research project. He pointed out that the two give different expectations to the users and therefore affect uptake. A research project is expected to end at some point and so the attitude of a user in terms of utilization will not be a long-term one. Yet in the case of a service, it is viewed as long-term and not given an expiry date in the mind of the user and so they are likely to look it up even when they have not used it in a while.

Respondents referred to the physical location of the service as part of its visibility. They referred to the location of the service being at a prominent university as a factor that gave it more and automatic visibility that other locations might not be able to. However, one policy-maker felt that location at a university might also have been a cause of inadequate visibility, as it is not a place that most potential users access often. He preferred the location being where many of the users are, for example in this case, at the MoH.

One researcher noted that continuity of activities was important for the uptake of a RRS. She noted this in reference to the service running as a project. She noted that activities that are seen to spike and then wane out before spiking again affect uptake. She noted that researchers should endeavor to keep the momentum of activities consistent otherwise in times when they wane, utilization of the service may be affected negatively to the extent of not recovering when activities spiked again.

#### Integrity

All respondents pointed towards integrity, credibility, and trustworthiness as being important in some way for the uptake of the RRS. For the RRS, this integrity was measured on several things like who was running the service and who their partners were. Other factors determining credibility included the location of the service – some places were seen as commanding more credibility than others; for example, one policy-maker felt that the RRS service being hosted by Makerere University gave it credibility. He noted that this location gave it a sign of neutrality and being incorruptible. However the service’s location at the University was also pointed out as one to inspire mistrust in some groups of policy-makers. One policy-maker noted that being located at Makerere University made the service look like an ‘outsider’ to the policy process, one where the academics had very little understanding of how the ‘real’ world functions, preferring that it be located in the policy-making institution if it was to be taken up more easily. One researcher was in agreement with this idea:

*“… the university is not a service provider for health issues, the university is an academic center, it is education, its interest in the service is researching on the service, educating for the service, but not a direct provider of services and so if we are talking about services specifically, probably it should not be in the university. But if we are talking about the academics of a RRS just like the academics around patient care that is university business” (*Researcher).

In addition, one researcher noted the need for full disclosure about the preparation process and how one arrived at the final summarized evidence, to prove credibility.

*“… having a systematic and transparent process for responding, where it’s clear what was done, how it was done or not done, when it was done …” (*Researcher).

#### Networks and Relationships

The relationships that the service builds with its stakeholders are important and are a determinant of its being taken up by potential users. Such stakeholders include users and other players in the research and policy-making arenas. How relationships improve uptake included continued interaction, collaborations on the supply side, use of ‘champions’ and advocacy.

When considering the potential users, one researcher working on the service noted that it was important for researchers on the RRS supply side to find a careful balance between an institutionalized system and a personal relationship, noting that both were important for uptake. She noted that it was important to ensure an institutionalized service which would ensure continuity at all times and breaks in service that might affect uptake, while it was also important to have a closer interaction with the policy-makers to understand their day to day needs, the contexts in which they work and to build trust.

One policy-maker also pointed out the need for the service to ensure interaction allowing for the users to work through framing what the issue is with the researcher.

*“Sometimes we need help to express the question or some issue and come to a reasonable place but if the service presents a robot to take my question, will it help me to think through my question? We need to discuss …”* (Policy-maker).

In line with the interaction, it was important that the researchers maintained their credible outlook with the policy-makers and stakeholders. One researcher remarked:

*“… can you sustain your reputation as a credible, neutral and independent purveyor of the best available evidence and not a group that takes sides, hmm you know, does some things outside the public eye like doing some reports that are not put in the public domain. There is a set of things that you have established for your credibility that if you were to lose them, there would be consequences …”* (Researcher).

Important relationships were not only with users but with other stakeholders too. One policy-maker noted that it is important to maintain a good relationship while forming collaborations. Such relationships communicate to the users too. He noted that collaboration with others, for example support units at the MoH should be seen as complementary not competitive. Researchers also noted the need for the service to make relationships that are supportive to researchers, as these researchers are important sources of the research evidence and for reviewing the products of the service.

Another important relationship pointed out as important for uptake was that between policy-makers as peers. This was deemed important because of the tendency of the RRS to use policy-makers to advocate for and sensitize their peers about it. Such policy-makers are referred to as champions. Using peers who have a great relationship with their colleagues will lead to better uptake than those who understand the concept but do not relate well with their colleagues.

In terms of relating, one policy-maker noted a relationship that is created when the RRS is involved in regular advocacy and sends regular information to its potential users despite supplying policy briefs and evidence summaries. Providing the service needed additional advocacy for it, its products and the messages out of the products. Without this additional advocacy, uptake of the service, its products and the messages relayed by the products is still not automatic and may be slow. He gave an example:

*“For example, when the service sends a weekly reminder like an email that has say a recent brief, although it is like advertising, it creates some close relationship and as a user I can’t forget. It’s like when you begin to hum the song you hear all the time and some relationship is automatically created”* (Policy-maker).

### Environmental Factors

#### Political Will

Researchers noted that the work of the RRS would be taken up even more easily by users if it had explicit support from high level or influential players in the policy-making world. They noted that not only does this increase awareness and curiosity about it, it also gives it a sense of legitimacy. One researcher who was involved in the RRS in Uganda before piloting another RRS in another country noted as below:

*“We had entry, the health minister’s advisor and the director of research were part of our activities and drummed up support for us. Some people took it up because of the advisor … I think also in Zambia, there was traction because of [the minister]. You also gave an example, where … was it the head of a [technical working group], halted the work of the group to wait for you to return from a meeting so that they could consult you …”* (Researcher).

*“… In the meeting the director asked them, what is different you would be offering me that those people in Makerere (in reference to the RRS) are not. Afterwards, everyone wanted to know who the Makerere people were …”* (Researcher).

#### Health System Policy and Decision-Making Infrastructure

One researcher and two policy-makers noted that the *set-up of the policy and decision-making systems or bodies* was important for the uptake of mechanisms that support evidence to policy activities. They noted that these form a culture or an environment that compels policy-makers to use evidence for their decisions and in a bid to do this, they end up using the available mechanisms including the RRS.

*“Some systems are made in such a way that either you are forced to do KT or some activities towards it and to prove that you have done so. For example, I have heard that in Canada if you are proposing a new course of action, it is a requirement for you to show how you have consulted the evidence, I think including research. In such a system, there is no two ways about it …”* (Researcher).

They noted that if there were clear steps incorporated in the decision-making process where evidence is expected to be considered, these would also lead to a culture that icreases the relevance and therefore uptake of RRSs and similar entities. Furthermore, they noted that if the entity is provided for or openly recognized by the government as a part of the system, its uptake is more guaranteed or improved.

*“I consider governments that provide supportive units, which are recognized by the government. Look at units like NICE [National Institute for Health and Care Excellence] in the United Kingdom, it is encouraging. I think if your unit was adopted and recognized by the government, it would be hard to ignore”* (Policy-maker).

## Discussion

This study aimed to use a process evaluation to determine the contextual factors that affect uptake of a RRS designed to meet policy-makers’ urgent needs for evidence in Uganda. We identified three thematic categories: internal and external factors, that is, factors that the RRS has control over and those that it only partially does, respectively. In addition there were environmental factors that the service has no control over. Under the internal factors were, the sub themes of the design and resources while under the external factors were the service’s visibility, integrity and relationships. The environmental factors included political will and the health system’s policy-making infrastructure.

### Findings in Relation to Other Findings

The findings here are reflective of the concepts of the systems theory.^27^ A basic element in the system’s theory is interaction, which is generated from the behavior of its different entities, when they each play their role. When these several interactions become a set of interrelations, they are considered a system. The elements identified in this study do formulate an interactive and dynamic system in which decisions are continuously made eventually leading to an output, an environment that affects the uptake of the RRS. The system is integrative involving values and norms, culture and behaviour, governance and processes, and a lot more. The system includes all forms of formal and informal processes and structures. These are important for its survival and uptake, favouring it or obstructing it.

In the design and resources of a RRS, we note the value of the supply side human resource. In their research on the feasibility of RRSs in LMICs, Healy and colleagues emphasized that RRSs might not be feasible in the absence of individuals readily and reliably available to receive and respond to policy-makers needs.^28^ In addition to the design, the final innovation or service as presented to the users will affect how it is perceived especially in line with the culture, norms and behavior of the users. Two studies including a meta-analysis of 75 studies concluded that three characteristics are consistently significant for the uptake of an innovation: relative advantage (meeting an obvious gap or need), compatibility with values and norms, and minimal complexity.^20,29^

Designing an innovation is not possible without resources of different kinds. A balance in quality and quantity of these ensures an effective and efficient service. For example, a right balance between the number and qualification and skills of the supply side human resources is very crucial for the innovation as an entity and output.^30^ Furthermore, the right skill mix, that is, both technical and non-technical is vital. The resource-based view (RBV) as a basis for the competitive advantage of an innovation treats the innovation as a bundle of resources of different kinds and that these which are organizationally internal influence the success of an enterprise.^30^ On resources, we noted with surprise though that respondents in this study did not mention research as one of the resources vital for its uptake. It may be that they took it for granted as available and therefore a basic for the service.

Increased visibility through different strategies influences uptake. Bower notes that theoretically, external influences on visibility such as promotion and marketing affect adoption behavior.^20^ However a study commissioned by the Rand Corporation found little evidence of the effect of promotions.^20^ In fact although promotions are often mentioned as drivers of adoption of innovations, there is very little empirical work that demonstrates its independent effect. Advocacy on the other hand that is guaranteed for the service and its products or messages through channels like the “champions” is a proven strategy to increase uptake of an innovation.^31^ Everett notes the need for ‘observability’ which may tally with our finding of visibility. Aside from the obvious awareness of the innovation that visibility creates, it also stimulates peer discussion of a new idea, as friends and colleagues of an adopter often request innovation-evaluation information about it.^29^

Relationships are one of the demand factors several scholars cite in their work about determinants of uptake, noting that there is network effects from an innovation.^9^ Bower notes the importance of social pressure via activated peer group networks. He notes that practitioners and hospital managers acquire adoption-relevant information about an innovation through informal contact with their peers. These peers who are generally early adopters may be looked at as “champions” of the innovation and their dissemination activities are referred to as “epidemic effects via activated peer group networks.”^32^ In close relation to relationships, is integrity. While, integrity leads to enhanced relations between users and producers of evidence, integrity and innovation are intuitively connected with improving overall performance, and the sustainability of that performance.^33^

The study of complex systems points towards the fact that relationships between components give rise to the collective behavior of a system, and how that system interacts and forms relationships with its environment.^34^ The components identified in the system that eventually leads to uptake of RRSs are unable to eventually do this each on their own. They can only eventually lead to uptake by interacting with each other. For example, if one has more resources they are able to improve the design and an improved design may in turn attract more resources. Or, a better design may increase the visibility of the service which in turn enhances relationships and networks, which attract more resources which lead back to a better design.

### Strengths

At the time of this research, we were not aware of any other efforts towards an evaluation of an RRS in a low-income country. The use of this particular evaluative approach is a strength as it does not only consider the final output but looks at the process and what goes into it. This is important as it is known that not all components of a program contribute to its success or outputs equally. Knowing which ones are vital at what point is important to create efficient use of limited resources.

### Implications for policy

We have presented here factors that affect the uptake of the RRS in Uganda for decision-making. Research elsewhere has proven their feasibility and the fact that they are valued by policy-makers. For health systems practitioners considering these, knowing what factors will affect uptake and therefore modify these within their contexts is important to ensure efficient use and successful utilization if the services. In addition, we have presented environmental factors that would help practitioners determine whether it is worth it starting a service or whether their initial efforts should be focused on advocating for a better environment in which they will practice.

### Implications for Research

In this research, we explored the contextual factors affecting the uptake of RRSs using a process evaluation. This does not take into account several things including the longer term outcomes of this process per se which is important and future research would be vital in informing the evidence gap still present. Furthermore, more evaluations in similar and other setting will provide a deeper understanding of these contextual factors for practitioners considering RRSs in their settings.

## Conclusion

RRSs are an innovation that aims to support the policy and decision-making process providing relevant and timely research evidence when it is needed. However, this can only happen if these mechanisms are fully adopted and utilized. There are modifiable internal and external factors of such a mechanism that affect its uptake. When health systems managers and KT experts know and understand these contextual factors, they are able to influence the extent to which, and the speed at which the mechanisms are adopted and furthermore keep this adoption sustained.

## Acknowledgements

This work is supported on a doctoral programme with funding from the International Development Research Centre, Canada under the International Research Chairs Initiative. The African Doctoral Dissertation Research Fellowship has also provided partial funding to enable this work. Work to set up the RRS was done under the SURE for Policy in African Health Systems project, funded by the European Commission’s seventh Framework Programme, grant agreement No. 222881. We acknowledge Prof. John Lavis and Dr. Andy Oxman who provided guidance on drafts of this manuscript.

## Ethical issues

Ethical clearance for this study was obtained from the School of Medicine, Research Ethics Committee of the College of Health Sciences, Makerere University, Kampala, Uganda.

## Competing interests

The authors declare they have no competing interests.

## Authors’ contributions

RM participated in conceiving the study, designing and implementing it. She also participated in data analysis and drafted the manuscript. NKS participated in the conception and design. He also participated in data analysis and reviewing iterations of the manuscript. Both authors read drafts of the manuscript and approved the final manuscript.

## Authors’ affiliations

^1^Clinical Epidemiology Unit, School of Medicine, College of Health Sciences, Makerere University, Kampala, Uganda. ^2^Regional East Africa Community Health Policy Initiative (REACH-PI) (Uganda Country Node), Makerere University, Kampala, Uganda. ^3^Department of Medicine, School of Medicine, College of Health Sciences, Makerere University, Kampala, Uganda.

## Supplematary Materials

Supplementary File 1

Supplementary File 2 Supplementary files 1 and 2 contain the questionnaire and interview guide, respectively.

## Key messages

**Implications for policy makers**

- We identify five subthemes of modifiable contextual factors affecting the uptake of a rapid response service (RRS) aimed to meet policy-makers’ needs for timely and relevant research evidence for urgent decision and policy-making.
- These findings are applicable in any setting especially those similar to Uganda. The factors would however need to be modified to meet the specific contexts in which they are to be applied.
- There are factors the practitioner would have full control over and those over which he or she would have only partial control.
- Knowing these factors helps practitioners leverage the usually limited resources for the system may have to develop and sustain an acceptable and valued RRS.

**Implications for public**

Decision-makers and health system managers are often faced with situations in which they need make decisions urgently and may need research evidence to support that process. They however are often faced with barriers in accessing and using that research evidence. Rapid response services (RRSs) help to provide the decision-makers with timely and relevant research evidence of high quality. What our research shows is factors that help to ensure the success of one such service, in terms of its uptake by potential users. It is important that such a service succeeds as this will improve the use of evidence for decisions. Using evidence such as that provided on such a service has been shown to lead to better decisions that ensure equity and efficient allocation of resources, which in turn improves health service delivery and improves health outcomes.
