# Supplementary material for: A Process Evaluation to Assess Contextual Factors Associated With the Uptake of a Rapid Response Service to Support Health Systems’ Decision-Making in Uganda
Source: Int J Health Policy Manag. 2017 Feb 4;6(10):561–71. doi: 10.15171/ijhpm.2017.04 (PMC5627784; doi:10.15171/ijhpm.2017.04)
Supplement: Supplementary File 1 [file ijhpm-6-561-s001.pdf]

IJHPM
International Journal of Health Policy and Management

Dear respondent,

Thank you for agreeing to be interviewed for this study and for taking the time to respond to these questions. Your participation is invaluable to the understanding of what makes stakeholders utilize (or fail to), rapid response mechanisms that support urgent policy and decision making.

A rapid response mechanism is a knowledge translation strategy designed to receive and respond to urgent requests for evidence about health systems during the decision and policy-making process. These provide evidence that is already appraised, contextualized and summarized to ease its use. Such a service exists in Uganda and you have been a part of it, either as a user or as someone quite conversant with how it operates.

The aim of this study is to determine the contextual factors that affect the uptake and utilization of a rapid response mechanism in Uganda.

Specifically, we are interested in your personal opinions regarding the design, set up and operation of rapid response services in Uganda. Such would include their structure and their activities for identified components. There are no correct or incorrect responses; we are merely interested in your personal point of view. You will be led through a series of questions that will help explore each of the components of the mechanism.

The average time of this interview is estimated at 30 minutes. The interviewer will enter the date and your subject identification number in the designated spaces on the questionnaire. Your subject Identification number is needed for follow-up surveys. However, all responses to this survey are completely confidential. All identifying information will be removed from this questionnaire and destroyed as soon as all data has been collected. Please be assured that the information you provide in this study will have **no effect on your usual work**.

Thank you for your participation in this study.

Process evaluation to determine the contextual factors that affect the uptake and utilization of a rapid response mechanism in Uganda

Date: ____________________

Subject Identification number: ______________________________

For a rapid response service to be taken up and utilized by users, what do you see the following components of the service looking like?

Components	Questions	Responses	
		Activity	Structural
**Service**	• **Discuss what such a service should be like and why?**<br>• **How should it be established?**		
**Goals and Objectives**	• **Discuss what the service's goals and objectives should be?**		
**Client/Target group**	**Discuss with reasons what the target group of this service should be.**<br>**Example of prompt questions:**<br>• Who should use such a service?<br>• What should the demographic characteristics of users be?<br>• What factors should they have in common?<br>• What percentage of each user group should be reached? Does everyone have to be reached? Are there priority groups?<br>• How should users be recruited or offered the program?<br>• Who should not use such a service?		
**Personnel**	**Who in your opinion should work on such a service?**<br>**Example of prompt questions:**<br>• What characteristics should they have?<br>• What qualifications should they have?<br>• How many staff should be on the service?<br>• How should recruitment be done?<br>• How should staff be trained?<br>• How should staff be remunerated/compensated?<br>• How long should staff work on such a service?		
**Equipment**	**What sort of equipment should be on this kind of service?**<br>**Example of prompt questions:**<br>• What software should such a service use?<br>• How should this equipment be procured?<br>• How should this equipment be maintained?<br>• Who should provide the software and how?<br>• Who should provide the maintenance and how?		
**Finances**	**How should the service be funded?**<br>**Example of prompt questions:**<br>• How should the budget be prepared?<br>• Should the service provide audited accountability on a regular basis?<br>• What should the annual budget of the service be or cover?<br>• What are the items funds should be spent on?<br>• Who should be the accounting person on the service?<br>• What should the process for acquisition of needs be like?		
**Operations**<br>**(List the different activities)**	**Describe what the operations of such a service should be like.**<br>**Example of prompt questions:**<br>• List the envisioned activities and how often they should be done?<br>• What do you see as the potential enablers and barriers for the operations and activities listed/mentioned?<br>• How should efficiency be measured?<br>• How should the reporting mechanisms be set?<br>• How should such a service collaborate with other peer services and organizations?		
**Products**	**What (different) products would you expect from such a service?**<br>**Example of prompt questions:**<br>• Who would prepare these products?<br>• Should there be a standard preparation format for the products?<br>• How should the standard preparation format be developed (if yes above)?<br>• How should the products delivered/disseminated?<br>• On average how many should be delivered/disseminated annually?<br>• Should the products be monitored/evaluated? How? By who? How often?<br>• How can the products be made accessible are the products to the particular/other clients?<br>• How should feedback mechanisms about the products be set up?<br>• Who should own the products?		
**Others**	**Discuss your envisioned M&E policy and plan for such a service**		
	**What made (would make) you use, return to use or recommend to someone else such a service?**		

Respondent bio data

1. Gender ☐ Male ☐ Female

2. Age group
   - < 30 years ☐
   - 30 – 39 years ☐
   - 40 – 49 years ☐
   - 50 – 59 years ☐
   - 60 – 69 years ☐
   - 70 and above ☐
3. Organization or affiliation --

4. Current position --

5. Years in position
   - < 5 years ☐
   - 5–10 years ☐
   - 11–15 years ☐
   - 16–20 years ☐
   - Over 20 years ☐
6. Researcher/policy-maker (*circle one that applies most to you)*

7. Years as researcher/policymaker
   - < 5 years ☐
   - 5–10 years ☐
   - 11–15 years ☐
   - 16–20 years ☐
   - Over 20 years ☐
8. Have you used the Cameroonian rapid response service before?
   ☐ Yes ☐ No

   a. If yes, have you used the service more than once?
   ☐ Yes ☐ No

   b. Have you recommended the service to anyone after you used it?
   ☐ Yes ☐ No
