# Supplementary material for: A Process Evaluation to Assess Contextual Factors Associated With the Uptake of a Rapid Response Service to Support Health Systems’ Decision-Making in Uganda
Source: Int J Health Policy Manag. 2017 Feb 4;6(10):561–71. doi: 10.15171/ijhpm.2017.04 (PMC5627784; doi:10.15171/ijhpm.2017.04)
Supplement: Supplementary File 2 — Supplementary files 1 and 2 contain the questionnaire and interview guide, respectively. [file ijhpm-6-561-s002.pdf]

IJHPM
International Journal of Health Policy and Management

12.1: Appendix I: Key informant Interview guide for study II and III
A case study of a rapid response service to meet health policymakers' urgent needs for research evidence about health systems in a low income setting (Uganda)
(Use alongside questionnaire)

Hullo,
My name is Rhona Mijumbi and I am the Principal Investigator on this research that is trying to describe the process of establishing the REACH rapid response service at Makerere University. The research is expected to inform researchers or/and policymakers considering similar initiatives. The study was approved by the research and ethics review board in the School of Medicine at the College of Health Sciences, Makerere University. You have been chosen as a key informant because I believe you were involved in the process of establishing a similar service. Your insights in what took place with your service in relation with the REACH rapid response service in Uganda will go a long way in improving our knowledge about such initiatives.
If you do agree to take part in this interview, I will ask you to sign the consent form provided and I will proceed to ask you a few questions. This interview is likely to take about 30-45 minutes.

Name: ……………………………………………………………………………………………………

Organization: …………………………………………………………………………………………..

Position: ……………………………………………………………………………………………………..

Researcher/Policy maker/KT specialist/Other (specify) …………………………………………
(Circle one that applies most) Ask if they have not filled this on the consent form

1. For how long have you been (were you) in contact with the REACH rapid response service?

2. How did you come to learn about the REACH rapid response service?

3. What interested you about the REACH rapid response service at the time?

4. How did you contact the REACH rapid response service?

5. What did you learn from the REACH rapid response service that you felt was instrumental to you and/or your work?
    a. How did you apply this?

(If policymaker, jump to question 10)

For those involved in setting up the RRS or a similar initiative

6. For how long have you been (were you) a part or in contact with the service in your country/organization?

7. At what stage were you involved? (*Tick all that apply*)
    a. Idea gestation
    b. Planning
    c. Piloting
    d. Scaling up
    e. Institutionalization

8. What was/is your role in each of the above steps of the establishing of your *rapid response service*?
    Prompt
    a. Why was/is the role pertinent?
    b. How did/do you execute it?

9. How do you think you affect(ed) your service?
   (Or describe your experience working/dealing with the service)

10. What was/is your understanding of the service in terms of:
    a. Structure
    b. Function/Activities

** *(Use the questionnaire to explore the different components)*

11. What do you see as the main components of such a service when at its best?
    Prompt by stage

12. In your opinion have these (in #6 above) been achieved
    Prompt by stage
    i. with the REACH rapid response service in Uganda?
    ii. with your service?
    a. If yes, why?
    b. If no, why not?

13. Briefly describe the **policy environment** in your country as it may affect the functioning of the RRS

14. Briefly describe the **knowledge translation environment** in your country as it may affect the functioning of the RRS

15. Does the service fit the above two environments?
    a. If yes, why and how?
    b. If no, why not?

16. What do you see as having contributed to the earlier success/failure of the program?

17. What do you think will contribute to the continued/eventual success/failure of the program?

Thank you for your time.
